# A pharmacokinetic and pharmacodynamic investigation of Modufolin^®^ compared to Isovorin^®^ after single dose intravenous administration to patients with colon cancer: a randomized study

**DOI:** 10.1007/s00280-014-2611-9

**Published:** 2014-10-24

**Authors:** Yvonne Wettergren, Helena Taflin, Elisabeth Odin, Karl Kodeda, Kristoffer Derwinger

**Affiliations:** Department of Surgery, Institute of Clinical Sciences, The Sahlgrenska Academy at University of Gothenburg, Gothenburg, Sweden

**Keywords:** Colon cancer, 5-FU-based chemotherapy, Levo-leucovorin, Methylenetetrahydrofolate, CH_2_THF, Tissue and plasma folates, LC-MS/MS

## Abstract

**Purpose:**

Leucovorin is commonly used as folate supplement in 5-fluorouracil-based chemotherapy, but needs to be converted to active 5,10-methylenetetrahydrofolate (methyleneTHF) intracellularly. This provides for interindividual differences. MethyleneTHF has recently been developed into the stable, distributable drug, Modufolin^®^. The aim was to compare the concentration of folate metabolites in tumor, mucosa, and plasma of patients with colon cancer after administration of Modufolin^®^ or Isovorin^®^ (levo-leucovorin).

**Methods:**

Thirty-two patients scheduled for colon resection were randomized to receive Modufolin^®^ or Isovorin^®^ at dosage of 60 or 200 mg/m^2^. The study drug was given as one i.v. bolus injection after anesthesia. Plasma was collected for pharmacokinetic (PK) analysis before, during, and after surgery. Tissue biopsies were collected at surgery. Folate metabolites were analyzed by LC-MS/MS.

**Results:**

MethyleneTHF and THF concentrations were significantly higher in mucosa (*p* < 0.01, both dosages) and tumors (*p* < 0.01, 200 mg/m^2^) after Modufolin^®^ as compared to Isovorin^®^ administration. The results correlated with PK observations. The Modufolin^®^ to Isovorin^®^ *C*_max_ ratio for methyleneTHF was 113 at 200 mg/m^2^ and 52 at 60 mg/m^2^; the AUC_last_ ratios were 17 and 9, respectively. The THF plasma concentrations were also higher after Modufolin^®^ administration (*C*_max_ ratio 23, AUC_last_ ratio 13 at 200 mg/m^2^; *C*_max_ ratio 15, AUC_last_ ratio 11 at 60 mg/m^2^).

**Conclusion:**

Modufolin^®^ administration resulted in significantly higher methyleneTHF levels than Isovorin^®^ and may potentially increase the efficacy of 5-fluorouracil-based chemotherapy. The results encourage further evaluation of Modufolin^®^ as a substitute to Isovorin^®^ including the potential clinical benefits.

**Electronic supplementary material:**

The online version of this article (doi:10.1007/s00280-014-2611-9) contains supplementary material, which is available to authorized users.

## Introduction

**Fig. 1:**
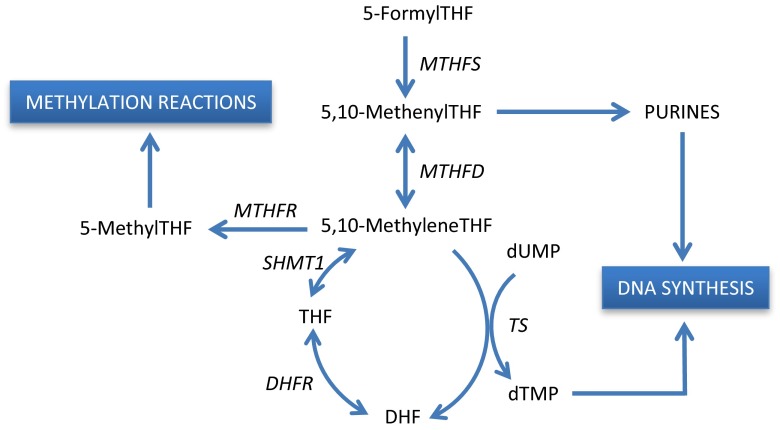
A simplified overview of the folate metabolism. Within the cells, Modufolin^®^ (5,10-methyleneTHF) can be used directly as a methyl donor in the synthesis of dTMP from dUMP. The reaction is catalyzed by the enzyme thymidylate synthase (TS). Isovorin^®^ (5-formylTHF), on the other hand, needs to be converted in two steps to methyleneTHF. Treatment with 5-FU inhibits the synthesis of dTMP through the formation of FdUMP, which binds TS. *DHF* dihydrofolate, *DHFR* dihydrofolate reductase, *SHMT1* serine hydroxymethyltransferase 1, *MTHFR* methylenetetrahydrofolate reductase, *MTHFD* methylenetetrahydrofolate dehydrogenase, *MTHFS* methenyltetrahydrofolate synthetase

Folates are naturally occurring forms of the soluble vitamin B9. They are fundamental to the cell by serving as one-carbon donors in the synthesis of purines and the pyrimidine deoxythymidine monophosphate (dTMP) [1]. Imbalance of the folate metabolism is suggested to affect several cellular processes, as folates are vital both for DNA synthesis and repair, as well as gene regulation by DNA methylation. Intracellular reduced folates exist as a pool of several interconvertible forms, which are involved at different stages in folate dependent and interconnected metabolic pathways (Fig. 1). The understanding of the underlying mechanisms with respect to transport, communication, and regulation among these metabolic pathways and networks is still incomplete and under investigation [2–4].

Folates can be used to modulate the action of the commonly used drug 5-fluorouracil (5-FU) [5, 6]. The original response rate of 5-FU in colorectal tumors, given as a monotherapy, was only around 10 %. By adding the stable, reduced form 5-formyltetrahydrofolate (formylTHF) i.e., leucovorin (LV), the tumor response rate was improved to 21 % as shown in a meta-analysis [5]. Intracellularly, LV needs to be converted to the active metabolite 5,10-methylenetetrahydrofolate (methyleneTHF). This metabolite then forms a ternary complex with deoxyuridine monophosphate (dUMP) and the target enzyme thymidylate synthase (TS) in a reaction where dUMP is converted to dTMP [6, 7]. The reaction is inhibited when the fluorinated metabolite of 5-FU, FdUMP, binds the complex instead of dUMP [8, 9].

Thus, LV has no antitumoral effect on its own, but enhances the effect of 5-FU by providing methyleneTHF in abundance, which stabilizes the ternary complex [10]. The inhibition of TS impairs production of dTMP needed both in DNA synthesis and DNA repair. The inhibition will have most impact on cells with a high proliferation rate, such as tumor epithelial cells. As a consequence, the DNA synthesis in the cells is suppressed which may lead to cell death due to apoptosis. Insufficient cellular levels of methyleneTHF could be an important factor behind the lack of TS inhibition during 5-FU chemotherapy. An enhanced 5-FU effect would reduce the required quantities of toxic metabolites and, thus, the toxic side-effects during treatment would theoretically be less. The pharmacokinetics of LV metabolites as a function of intravenously given dosage has been described by Priest et al. [11].

The need for metabolic activation of LV could provide for interindividual differences in its utilization that could affect the potential benefit gained from the LV addition. Previous results, including findings of our own research group [12], have showed that the variation in folate levels in tumor and mucosa tissues between patients is significant. The continued advances in genetics and metabolomics provide new possibilities for advanced studies of the folate metabolism and enables greater understanding of the interplay between folates and enzymes targeted by different chemotherapeutic drugs.

As methyleneTHF is the only natural folate that directly binds the ternary complex, its usage could have advantages compared to LV. However, as a substance, it is very sensitive to oxidation. The stability, production, and administration issues have recently been resolved and methyleneTHF has been developed into the stable drug Modufolin^®^. The aim of the present study was to gain an understanding of how a single bolus injection of Modufolin^®^ affects the concentration of different folate metabolites, including methyleneTHF, in tumor tissue, adjacent mucosa, and plasma as compared to Isovorin^®^ (levo-leucovorin) in patients with colon cancer using a highly sensitive liquid chromatography electrospray ionization tandem mass spectrometry (LC-MS/MS) method [13].

## Methods

### Patients

The study was performed as a randomized, single-blinded, phase I/II study, at a single center (Sahlgrenska University Hospital/Östra, Gothenburg, Sweden). Between September 2012 and July 2013, 32 patients scheduled for colon resection due to colon cancer were screened and asked for participation. The main inclusion criteria were: Age ≥18 years, ECOG performance status of 0–1 and resectable colon cancer/curative intent of surgery, whereas the main exclusion criteria were: Concurrent other antitumor therapy, other malignant disease, severe systemic disease, or medications which could influence homocysteine, folate, and vitamin B12 status, within 30 days of surgery. All included patients provided written informed consent. The study was done in accordance with the Declaration of Helsinki and adhered to the ICH Good Clinical Practice Guidelines. The protocol complied with local regulations and was approved by the Institutional Review Board and the Swedish National Competent Authority, Medical Products Agency (MPA). The study was also approved by the Regional Ethics Committee in Gothenburg (EPN).

### Randomization and masking

Included patients were randomly assigned (in a 1:1:1:1 ratio, with a block size of eight) to receive either Modufolin^®^ or Isovorin^®^ at a low (60 mg/m^2^) or high (200 mg/m^2^) dosage. Treatment group assignment was based on a computer-generated randomization list. Assignment to treatment was done in a consecutive order by means of randomization envelopes, which were opened by the study nurse preparing the study drug at the time point of study drug administration. The patient’s study identification number was used on the electronic case report form and as identifier on all samples.

The patients and the study team were masked with one exception; it was not possible to mask the study drug to the study nurse who prepared the bolus injection. The reason for this was that Modufolin^®^ is provided as a powder for injection that needs to be reconstituted in saline in connection with administration, whereas Isovorin^®^ is provided as a solution for injection. After reconstitution, Modufolin^®^ has a similar appearance as Isovorin^®^. However, all other involved study personnel were masked. The statistical and clinical study teams at the sponsor remained masked during the study. The study code was broken after declaration of clean file and database lock.

### Design and aim

The patients received one i.v. bolus injection of study drug after being anesthetized for surgery. Plasma samples were collected for pharmacokinetic (PK) analysis 5–20 days before surgery and on the day of surgery (after anesthetics and immediately prior to study drug administration) as well as 5, 10, 20, 40, 60, 90, 120, 180, 240, and 360 min after study drug administration. Plasma samples were also taken the day after surgery; at 24 h. End of study assessments were made 5 ± 2 days after surgery. A complete physical evaluation was done at inclusion and again at end of study participation. Adverse events (AEs) were assessed continuously to monitor safety aspects. The primary endpoint was tissue (tumor and adjacent mucosa) concentrations of methyleneTHF, THF, methylTHF, and formylTHF. Secondary endpoints consisted of PK parameters based on plasma concentrations of the same metabolites and correlations between plasma and tissue concentrations.

### Samples and handling

Biopsies from tumor and adjacent mucosa were collected during surgery. The macroscopically normal-appearing mucosa samples were taken at a distance of approximately 10 cm from the tumors. All samples were accrued in a standardized manner by dedicated research nurses. The actual biopsy collection time point and the time point when the circulation to the tumor was stopped were recorded. The time point for biopsy collection in relation to study drug administration and circulation stop time varied between patients due to individualized surgery procedures. The biopsy samples were snap-frozen in liquid nitrogen and stored at −80 °C until analyzed. Each sample was divided into several vials in order to avoid repeated freezing and thawing. The tissue concentrations of methyleneTHF, THF, methylTHF, and formylTHF were measured using an LC-MS/MS method as described previously [13]. To check for degradation of the four analytes, the intra- and interbatch variability was determined in tissue Q samples at low, medium, and high concentrations on the same day and over 5–8 days. The relative intra-batch standard deviation ranged from 4.8 to 10.2 % for all analytes, whereas the interbatch variability ranged from 4.9 to 20 %, with the highest variability found for methyleneTHF at the low Q sample concentration.

Plasma was obtained from the patients’ arm that was not used for study drug administration as close to the predefined time points as possible. The exact time points were recorded in the eCRF. Nominal sampling times were used for PK calculations as deviations from actual values were considered to be small (generally within ±20 %). A few time points in the beginning of the sampling period after dosing deviated >20 %, but this was considered to have small impact on the PK calculation outcome. The plasma samples were frozen, stored, and shipped at −80 °C to Charles River Laboratories, UK, where the plasma concentrations of methyleneTHF, THF, methylTHF, and formylTHF were analyzed using a validated LC-MS/MS method. In brief, samples were separated and quantified using a Poroshell 120 EC-C18 column (Agilent Technologies). The mobile phase consisted of a variable mixture of A (acetonitrile/formic acid), and B (water/formic acid) with a flow rate of 0.5 ml/min. The autosampler temperature was set to 4 °C and the column maintained at 60 °C. Transitions for analytes and ^13^C_5_-labelled internal standards were as shown in Table 1. Quantitation was done by comparison to a 9-point calibration curve (100–10,000 ng/ml) using the ratio of the analyte response to the internal standard plotted against the analyte injected. The mass spectrometer was run in the negative MRM mode. The reinjection stability was found to be 52 h for methyleneTHF, THF, and formylTHF, and 7 days for methylTHF, at 4 °C. Both tissue and plasma analyses were performed batch-wise.

**Table 1:** Folate analyte and internal standard transitions used for quantification of folates in plasma

Analyte/internal standard	Precursor ion (*m*/*z*)	Product ion (*m*/*z*)
MethyleneTHF	456.4	327.0
THF	444.2	315.0
MethylTHF	458.4	329.1
FormylTHF	472.1	343.1
^13^C_5_-MethyleneTHF	461.5	327.0
^13^C_5_-THF	449.2	315.1
^13^C_5_-MethylTHF	463.4	329.1
^13^C_5_-FormylTHF	477.1	343.1

### Statistical analysis and considerations

This was an exploratory proof of concept study, and the number of patients chosen was based on practical considerations and not on a statistical power calculation. Statistical analyses were done with SAS version 9.3 or JMP 10.0 (SAS Inc, Cary, USA). The significance level was set at 0.05.

An exploratory evaluation of the primary endpoint, i.e., tumor and mucosa concentrations of methyleneTHF, THF, methylTHF, and formylTHF was done, respectively, for differences between treatments using analysis of variance. The same evaluation was done for the secondary endpoint, PK parameters, in plasma. It was found that the assumptions of normality were violated, and therefore nonparametric methods (Proc GLM and Levene’s test) were applied to determine differences in variability between the treatment groups. The Kruskal–Wallis and Wilcoxon two-sample test (two-sided) were used to compare differences between treatments.

The PK/pharmacodynamic (PD) analyses were done including all patients who completed the trial without any major deviations from the protocol procedures. All included patients who received trial medication and from whom at least one measurement was obtained were assessed for safety. Pearson’s correlation coefficient was used as a measure of linear relationship between tumor and mucosa concentrations of methyleneTHF, THF, methylTHF, and formylTHF and between tissue concentration and plasma PK parameters of the same metabolites.

Plasma concentration versus time data was analyzed using individual profiles from patients by non-compartmental analysis (NCA) using Phoenix WinNonlin Professional^®^ version 6.3 (Pharsight Corporation, North Carolina, USA) according to internal procedure (SOP PKx101 version 1). In addition, graphical presentation of the PD and PK/PD data was done using the PhoenixWinNonlin Professional^®^ software and Microsoft Excel 2013. The area under the plasma concentration–time curve from time zero to the time of the last quantifiable concentration (AUC_last_) was used as PK comparator as well as the maximum observed plasma concentration (*C*_max_).

## Results

### Patients and safety

Demographic data of the included patients, as well as trial profile and treatment data, are presented in Table 2. One patient randomized to Isovorin^®^ (60 mg/m^2^) was withdrawn prior to administration of study drug due to an adverse reaction toward the administered epidural anesthesia. This patient was excluded from both per protocol and safety analyses. One patient each from the Modufolin^®^ (60 mg/m^2^) and the Isovorin^®^ (60 mg/m^2^) groups were excluded from per protocol analysis because the pathological evaluation of the specimens yielded the diagnosis adenoma and thus no malignant tissue was available for analysis. These two patients were included in the safety analysis.

**Table 2:** Patient demographics, trial profile, and treatment data of the randomized study patients shown by treatment arm

Treatment	Isovorin^®^		Modufolin^®^	
Dosage	60 mg/m^2^	200 mg/m^2^	60 mg/m^2^	200 mg/m^2^
Primary inclusion (*n*)	8	8	8	8
Safety population (*n*)	7^a^	8	8	8
Gender (M/F, *n*)	3/4	4/4	4/4	5/3
Mean age (range)	67 (41–82)	80 (66–93)	72 (57–89)	72 (48–88)
AEs (*n*)/patients (*n*)	1/1	4/4	9/5	10/4
SAEs (*n*)	1^b^	0	2^c,d^	2^e,f^
Per protocol population (*n*)	6^g^	8	7^g^	8
Tumor stage (I/II/III, *n*)	0/3/3	2/5/1	0/3/4	2/3/3
Tumor location (right/left, *n*)	3/3	6/2	4/3	5/3
Time to vessel ligation^h^	63 (35–110)	79 (37–124)	70 (37–105)	116 (30–140)
Time from ligation to biopsy^h^	50 (25–186)	40 (22–136)	38 (27–83)	44 (33–265)

^a^Excluded before drug administration due to reaction to anesthesia, ^b ^cardiac arrest, ^c ^pulmonary embolism, ^d ^myocardial ischemia, ^e ^wound dehiscence, ^f ^anastomotic leakage, ^g ^one patient excluded postoperatively as pathology report revealed adenoma, ^h ^time measurements in minutes with median (range) shown

The total number of AEs, including five serious adverse events (SAEs), reported from the study (safety population, *n* = 31) were 24, experienced by 14 patients. As shown in Table 2, one patient could experience many AEs on the same occasion. For example, one patient in the group who received 200 mg/m^2^ Modufolin^®^ suffered from an anastomotic leak 5 days after surgery and hence experienced six different AEs, e.g., pyrexia, abdominal pain and vomiting. One patient, randomized to Isovorin^®^ (60 mg/m^2^), died of a cardiac arrest 2 days after surgery. This patient was included in both per protocol and safety populations since the patient was not in violation of the study protocol during the collection of plasma and tissue samples. One more patient was lost in follow-up 20 months after surgery, cause of death unknown. Postoperative complications including wound dehiscence, anastomotic leakage, and pulmonary embolism were among other SAEs, as presented in Table 2. None of the AEs/SAEs were considered by the safety physician as having any suspected relationship with study treatment, i.e., neither with Modufolin^®^ nor with Isovorin^®^. No suspected unexpected serious adverse reaction (SUSARs) occurred in this study.

### Pharmacodynamics (per protocol population, *n* = 29)

**Fig. 2:**
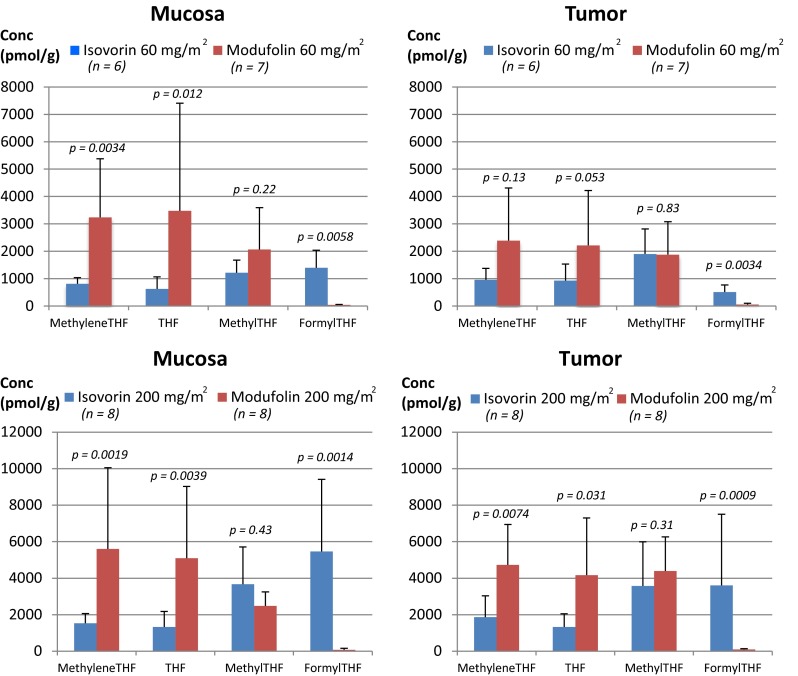
Comparison of mean concentrations (including SD) of methyleneTHF, THF, methylTHF, and formylTHF in mucosa and tumor after 60 or 200 mg/m^2^ Isovorin^®^ or Modufolin^®^ (*n* = 29, per protocol population)

The differences in magnitude and significance of the analyzed folate metabolites after Modufolin^®^ and Isovorin^®^ administration, respectively, are shown by drug, dose, and metabolite in Fig. 2 and Supplementary Table 1. Modufolin^®^ administration resulted in significantly higher mucosa concentrations of methyleneTHF (*p* < 0.01 at both dose levels) and THF (*p* < 0.05 at 60 mg/m^2^, *p* < 0.01 at 200 mg/m^2^) than did Isovorin^®^. Higher concentrations of methyleneTHF and THF were also observed in tumor tissue after Modufolin^®^ administration. The concentration difference in tumor was statistically significant in favor for Modufolin^®^ at 200 mg/m^2^ (*p* < 0.01 for methyleneTHF and *p* < 0.05 for THF), while significance was not reached at the lower dose level for methyleneTHF or THF. The methylTHF levels did not differ significantly according to treatment, neither in mucosa nor in tumor tissue. The formylTHF levels, on the other hand, were significantly higher in both mucosa and tumor tissue of patients treated with Isovorin^®^ as compared to Modufolin^®^, at both doses.

A statistically significant linear correlation of tumor and mucosa concentrations was confirmed for all studied folate metabolites after Modufolin^®^ (60 mg/m^2^) treatment. The correlation was significant for methyleneTHF (*r* = 0.90, *p* < 0.01), THF (*r* = 0.98, *p* < 0.001), methylTHF (*r* = 0.97, *p* < 0.001), and formylTHF (*r* = 0.76, *p* < 0.05). No correlation between tumor and mucosa was found for any of the metabolites after administration of 200 mg/m^2^ Modufolin^®^ or Isovorin^®^ (60 or 200 mg/m^2^).

### Pharmacokinetics (per protocol population, *n* = 29)

**Fig. 3:**
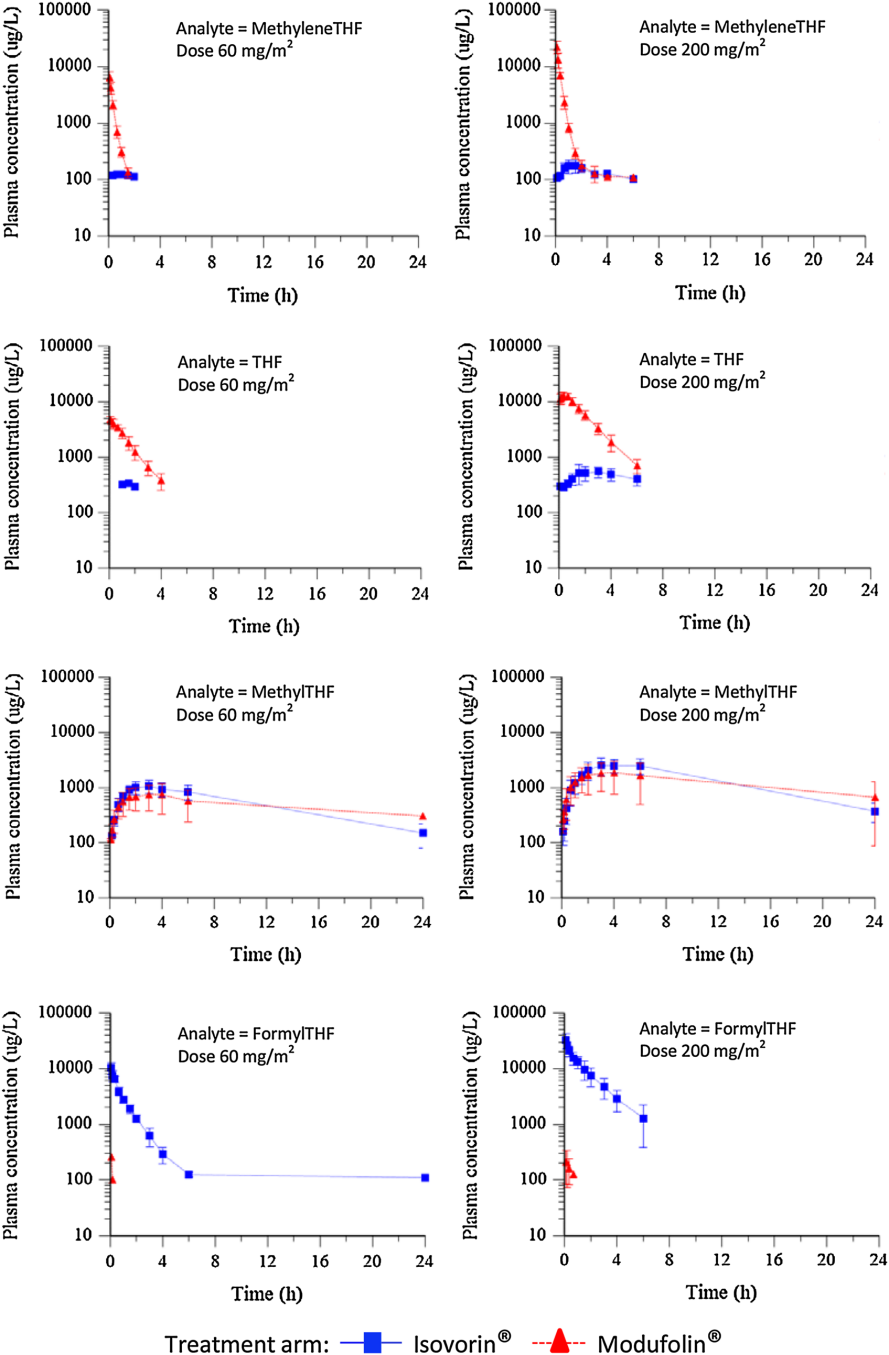
The mean plasma concentration and SD of methyleneTHF, THF, methylTHF, and formylTHF following 60 and 200 mg/m^2^ administration of Isovorin^®^ or Modufolin^®^ at different time points are presented for the per protocol population (*n* = 29)

The lower limit of quantification (LLOQ) for the folate metabolites in plasma ranged from 100–250 µg/L. The concentration of the folates in plasma samples collected 5–20 days before surgery, and on the day of surgery prior to drug administration, was below the LLOQ. The mean plasma concentrations of the folate metabolites versus time following Modufolin^®^ and Isovorin^®^ (60 and 200 mg/m^2^) treatments are presented in Fig. 3. The pharmacokinetics could not be fully evaluated for methyleneTHF and THF after Isovorin^®^ treatment or for formylTHF after Modufolin^®^ treatment because the major part of the obtained results for the assessed metabolites was below the LLOQ.

**Fig. 4:**
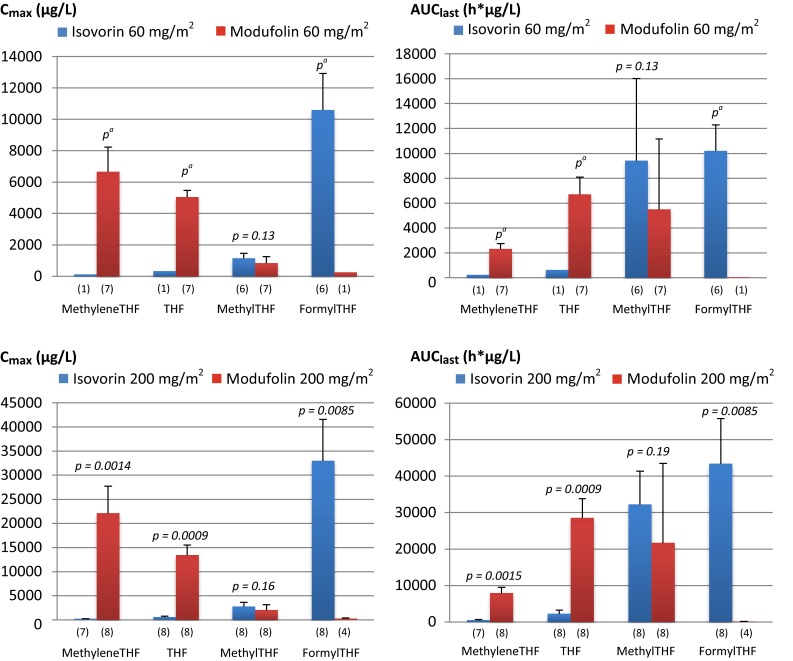
Comparison of mean *C*_max_ and AUC_last_ (including SD) of the folate metabolites methyleneTHF, THF, methylTHF, and formylTHF in plasma following administration of 60 mg/m^2^ Isovorin^®^ (*n* = 6), 200 mg/m^2^ of Isovorin^®^ (*n* = 8), 60 mg/m^2^ of Modufolin^®^ (*n* = 7), or 200 mg/m^2^ of Modufolin^®^ (*n* = 8). Numbers within parentheses indicate the number of plasma samples that had a folate concentration above the lower limit of quantification. ^a^Due to a limited number of observations, a *p* value could not be calculated

Administration of 200 mg/m^2^ Modufolin^®^ resulted in significantly higher plasma *C*_max_ and AUC_last_ of methyleneTHF (*p* < 0.01) and THF (*p* < 0.001) than did the same dose of Isovorin^®^ (Fig. 4). At the lower dosage, the resulting observations of both metabolites were below LLOQ for all patients except one in the Isovorin^®^ group, thus a statistical test of difference between treatments was not performed. The methyleneTHF *C*_max_ ratio of Modufolin^®^ to Isovorin^®^ was 113, and the AUC_last_ ratio was 17 at the 200 mg/m^2^ dose level. Performing similar calculations at the 60 mg/m^2^ dose level by comparing the mean methyleneTHF values for the Modufolin^®^ group, and the one single detectable observation for the Isovorin^®^ group resulted in an approximate *C*_max_ ratio of 52 and an AUC_last_ ratio of 9. The corresponding results for THF were a *C*_max_ ratio of 23 and an AUC_last_ ratio of 13 at the 200 mg/m^2^ dose level. At 60 mg/m^2^, the approximate THF *C*_max_ ratio was 15 and AUC_last_ 11.

As in tissues, the methylTHF levels in plasma were in the same order of magnitude after Modufolin^®^ and Isovorin^®^ administration. The calculated *C*_max_ and AUC_last_ ratios (Modufolin^®^ to Isovorin^®^) were 0.7 at both dose levels. Following treatment with Isovorin^®^, formylTHF was measured at high levels. Modufolin^®^ on the other hand resulted in only one formylTHF observation above LLOQ in the 60 mg/m^2^ group and four in the 200 mg/m^2^ group. Disregarding the relatively few observations for the Modufolin^®^ groups, the *C*_max_ and AUC_last_ Modufolin^®^ to Isovorin^®^ ratios were calculated to be 0.01 and 0.0017, respectively, at the high dose (*p* < 0.01), and 0.026 and 0.0053, respectively, at the low dose (*p* not applicable).

### PD and PK correlation

The presence of any linear correlations between tissue concentrations and plasma AUC_last_ observations of the different folate metabolites was explored for the different treatments. One statistically significant linear AUC_last_ correlation was identified in the Modufolin^®^ (60 mg/m^2^) group for methylTHF in mucosa (*r* = 0.76, *p* < 0.05). No further correlations were found for any metabolite or treatment.

### Time and demography

There was no statistical difference in folate levels related to age, gender, or tumor location. Neither was there any relation between folate levels and tumor staging and pathology although there was a trend of higher folate levels along with a worse differentiation grade. Patients who suffered SAEs had higher levels of THF in tumor tissue and mucosa (*p* < 0.05). The time between ligation of main blood vessels and removal of bowel specimen, thus sampling of biopsy, did not affect the folate levels. The time from i.v. bolus of study drug until vessel ligation did relate to tissue levels of methyleneTHF (*p* < 0.05) and methylTHF (*p* < 0.01) with shorter time being associated with lower folate levels. This was not seen in mucosa.

## Discussion

The present study showed that significantly higher levels of methyleneTHF were obtained in both tissue biopsies and plasma after administration of Modufolin^®^ compared to Isovorin^®^ (Figs. 2, 3, 4). The finding could have implications in treatment of colorectal cancer as folate in the form of methyleneTHF is necessary in the formation of a ternary complex together with TS and the fluorinated metabolite of 5-FU, FdUMP [5–7]. The complex provides an inhibitory effect on a critical enzymatic pathway, which normally generates the necessary nucleotide substrates for DNA synthesis (Fig. 1). Thereby, the cell division cycle is impeded, and tumor cells can be triggered into apoptosis. While treatment efficacy can be difficult to assess in adjuvant settings, without visible tumor, several studies have confirmed that 5-FU-based chemotherapy improves both overall and disease-free survival for patients with stage III-disease [14]. Also, 5-FU-based chemotherapy has been shown to prolong overall survival in palliative settings [5]. The regimes have been duly updated with additions of antibodies or newer drugs such as oxaliplatin into more effective combination therapies [15, 16].

Although well established in use, the optimal LV dosage for 5-FU modulation is still not known. Different regimes in clinical practice worldwide use doses of LV from 60 to 500 mg/m^2^ [17–19]. In Nordic FLV therapy, 60 mg/m^2^ of LV is given as a bolus injection together with 500 mg/m^2^ of 5-FU [19]. Due to the low response rates and the omnipresent problem of toxicity all aspects on improvements would be of interest. The interest is not diminished by the fact that the drugs, in various doses and combinations, are commonly used at a great scale worldwide. One potential issue with LV to be addressed is the need for its metabolic activation. Growing insight in genomics and metabolomics leads to the suspicion that all individuals do not respond fully to LV as folate supplementation and, therefore, do not experience an optimal treatment effect [11, 20, 21]. Hence, one ambition has been to develop a distributable form of methyleneTHF, which could be used to bypass the enzymatic steps needed to convert LV to methyleneTHF. It has been argued that response to LV administration is associated with considerable intra- and interpatient PK variability that could be explained by differences in folate metabolism [21–24]. Administration of Modufolin^®^ instead of LV might circumvent such metabolic obstacles. Furthermore, an abundance of methyleneTHF in the tumor tissue may be generated by using Modufolin^®^, which might lead to an increased response to 5-FU-based chemotherapy by stabilization of the ternary complex [11, 25].

As shown in Fig. 2, significantly higher methyleneTHF and THF levels were seen in mucosa and tumor tissue after administration of 200 mg/m^2^ Modufolin^®^ compared to Isovorin^®^, as well as in mucosa at 60 mg/m^2^ Modufolin^®^. A difference was also seen in tumor tissue at 60 mg/m^2^ but it did not reach statistical significance, possibly due to low number of patients in the low dosage arms (Table 2). The higher levels of both methyleneTHF and THF in tumor and mucosa resulted most probably from the interconversion between these two metabolites [26]. Thus, the pool of THF + methyleneTHF in mucosa and tumor tissue was dramatically higher after Modufolin^®^, as compared to Isovorin treatment^®^. This means that the probability of reaching an optimal level of the cofactor methyleneTHF is higher after administration of Modufolin^®^ compared to Isovorin^®^. The high interpatient variability in folate concentrations observed in all groups may be linked to differences in the activity of enzymes involved in folate-associated pathways. For instance, folate polyglutamation, which is regulated by the enzymes folylpolyglutamate synthase and γ-glutamyl hydrogenase, is known to have a high impact on the folate status [27]. Genotype differences between individuals may also affect the tissue folate concentration. Polymorphisms in the gene methylenetetrahydrofolate reductase may be rate-limiting for the transition of methyleneTHF to methylTHF and might partly explain why the methylTHF levels did not differ according to the different treatments [12, 28–30]. As expected, the formylTHF levels were significantly higher in all tissues after Isovorin^®^ as compared to Modufolin^®^ administration. The reason for this presumption is that no formylTHF is generated from methyleneTHF due to the irreversible conversion of formylTHF to methenylTHF in the reaction catalyzed by the enzyme methenyltetrahydrofolate synthetase (Fig. 1). The minute amounts of formylTHF detected may result from a build-up of endogenous formylTHF due to the excessive amounts of methyleneTHF, added in the form of Modufolin^®^, which probably slow down the conversion rate of methenylTHF to methyleneTHF.

The present study included colon tumor tissue, which could emulate a neoadjuvant treatment situation or that of preoperative chemotherapy. The effect on metastasis tissue could reasonably be assumed to be similar in character but needs to be addressed and confirmed in clinical studies. The same theory could be applied to the adjuvant treatment setting, where however studies are difficult to perform as there usually is no visible tumor tissue, and the target is presumptive circulating tumor cells or micrometastasis. The exact mechanism of 5-FU-related toxicity is also not totally understood. Therefore, changes in folate supplementation could warrant safety studies that monitor treatment side-effects. There may also be a need to change dosages as well as the administration timing, given the pharmacokinetic properties of 5-FU.

Although none of the AEs/SAEs were considered to have any suspected relationship with study treatment, the study is limited in number of patients which makes it hard to draw conclusions with certainty. Still, patients who suffered SAEs had higher THF levels in tumor tissue and mucosa, and there was one cardiac event in the Modufolin^®^ arm. Therefore, it is reasonable to further address the issue of safety which, as always, needs to be weighed against desired antitumoral effects. While limited in size, the study was performed at a single center with all patients treated along the same guidelines and all samples obtained in a standardized manner by research nurses. The time elapsed from vessel ligation to collection of biopsy, with its’ wide range, could provide differences. The finding of an association between lower tissue levels of methyleneTHF (*p* < 0.05) and methylTHF (*p* < 0.01) and shorter time until vessel ligation is of importance. This association may relate to the pharmacokinetic properties as shown in Fig. 3. An early vessel ligation, which cuts off the blood supply, will inhibit further folate uptake resulting in lower tissue folate levels. In rectal cancer surgery or laparoscopic surgery, the ligation is commonly done early. In this study, however, the majority of the patients were subjected to open surgery with late ligation.

## Conclusion

Several studies have shown elevated concentrations of reduced folates in blood and tissue after LV administration; however, our study is the first to compare the results with those obtained after Modufolin^®^ administration. Significantly higher levels of methyleneTHF and THF were obtained with Modufolin^®^ as compared to Isovorin^®^. The higher levels could theoretically beneficially affect the formation of the ternary complex consisting of methyleneTHF, TS, and FdUMP and, thus, potentially increase the efficacy of 5-FU-based chemotherapy. The results stimulate to further evaluation of the impact of Modufolin^®^ as a substitute or complement to Isovorin^®^ on clinical outcome within different cancer therapy areas.

## Electronic supplementary material

Below is the link to the electronic supplementary material.

Supplementary material 1 (DOCX 13 kb)
