# Supplementary material for: A pharmacokinetic and pharmacodynamic investigation of Modufolin® compared to Isovorin® after single dose intravenous administration to patients with colon cancer: a randomized study
Source: Cancer Chemother Pharmacol. 2014 Oct 24;75(1):37–47. doi: 10.1007/s00280-014-2611-9 (PMC4281361; doi:10.1007/s00280-014-2611-9)
Supplement: Supplementary file 1 — Supplementary material 1 (DOCX 13 kb) [file 280_2014_2611_MOESM1_ESM.docx]

| Supplementary Table 1.  Folate metabolite concentrations in tumor and mucosa tissue by treatment arm. | | | | | | | |
| --- | --- | --- | --- | --- | --- | --- | --- |
|  | | Folate concentration (pmol/g_ww_)  mean±SD (min-max) | | | | | |
| Metabolite | Tissue | Isovorin^®^ | |  | Modufolin^®^ | | |
|  | | 60 mg/m^2^ | 200 mg/m^2^ |  | 60 mg/m^2^ | 200 mg/m^2^ | |
| MethyleneTHF | Tumor | 959±417 (461-1510) | 1870±1160 (848-3870) |  | 2390±1920 (1020-5420) | | 4730±2210 (2360-8860) |
|  | Mucosa | 816±218 (617-1080) | 1540±527 (972-2460) |  | 3240±2140 (1440-7430) | | 5610±4440 (2000-15800) |
| THF | Tumor | 933±598 (490-2130) | 1330±720 (521-2230) |  | 2220±2000 (774-6030) | | 4170±3130 (1080-10000) |
|  | Mucosa | 626±439 (199-1240) | 1330±852 (593-3240) |  | 3480±3930 (1030-11800) | | 5100±3930 (1790-11900) |
| MethylTHF | Tumor | 1900±918 (788-3540) | 3570±2420 (1160-8460) |  | 1880±1200 (871-4330) | | 4400±1860 (2090-7780) |
|  | Mucosa | 1220±455 (688-1820) | 3670±2040 (1360-6430) |  | 2070±1520 (1110-5410) | | 2490±765 (1490-3810) |
| FormylTHF | Tumor | 512±259 (333-1010) | 3610±3900 (870-12700) |  | 57.4±46.8 (22.7-152) | | 99.7±46.8 (44.7-189) |
|  | Mucosa | 1400±640 (626-2130) | 5460±3960 (189-13800) |  | 41.7±18.4 (22.4-77.4) | | 82.5±76.6 (43.6-271) |
